# Nutritional status and correlated socio-economic factors among preschool and school children in plantation communities, Sri Lanka

**DOI:** 10.1186/s12889-017-4311-y

**Published:** 2017-05-02

**Authors:** Lahiru Sandaruwan Galgamuwa, Devika Iddawela, Samath D. Dharmaratne, G.L.S. Galgamuwa

**Affiliations:** 1 Department of Parasitology, Faculty of Medicine, University of Peradeniya, Peradeniya, 20400 Sri Lanka; 2 Department of Community Medicine, Faculty of Medicine, University of Peradeniya, Peradeniya, 20400 Sri Lanka; 3 Institute for Health Metrics and Evaluation, Department of Global Health, School of Public Health, University of Washington, Box 357230, Seattle, Washington, WA 98195 USA

**Keywords:** Nutritional status, Socio-economic factors, Plantation communities, Sri Lanka

## Abstract

**Background:**

Child malnutrition is a major public health concern worldwide, leading to higher morbidity and mortality. It is mostly preventable through public health and economic development. The aim of the present study was to determine socio-economic factors associated with nutritional status among children in plantation communities, Sri Lanka.

**Methods:**

A cross-sectional study was performed among preschool and school going children in three rural communities of Sri Lanka from January to August 2014. Demographic and household characteristics were documented and anthropometric measurements were collected to calculate weight-for-age (WAZ), height-for-age (HAZ) and BMI-for-age (BAZ). Anthroplus, epiinfo and SPSS versions were used for the analysis of data.

**Results:**

A total of 547 children (aged 1–15 years, mean 7.0 ± 3.6 years, 53% female) participated in the study. 35.6%, 26.9% and 32.9% of children were underweight, stunting and wasting respectively. Undernutrition was more common in primary school children. Maternal employment, high number of siblings, high birth orders and female children were significantly associated with undernutrition among preschool children. Living in small houses, large number of family members, low monthly income and maternal employment were significantly associated with undernutrition among school children.

**Conclusions:**

Child undernutrition is a major public health concern in the plantation sector, Sri Lanka. Health education programs among the study population could be effective for solving the problem.

**Electronic supplementary material:**

The online version of this article (doi:10.1186/s12889-017-4311-y) contains supplementary material, which is available to authorized users.

## Background

Malnutrition is a major public health problem leading to child morbidity and an underlying cause for more than half of child deaths worldwide, particularly in low socioeconomic communities in developing countries [1, 2]. Insufficient dietary consumption and infectious diseases are major contributing factors for poor nutritional status of children [3, 4]. Young children are more prone to malnutrition because of high protein and energy needs and vulnerability of infections than adults [5]. Undernourished children are less productive of physical and mental development and increase susceptibility to infections and the risk of early deaths [6–8]. Malnutrition commonly has long lasting effects among children and adversely influences their adult logical and work capacity [9]. Anthropometric measurements are a cost-effective and reliable method to evaluate the health and nutrition status of the individuals and in a society [10]. To identify the actual priorities of child health within a society, it is necessary to determine the associations between individual variables and health-related factors in a child population.

Previous studies in Sri Lanka reported that undernutrition among children is still a major health problem associated with poor sanitation and personal hygiene, low socioeconomic status, overcrowding and low educated parents [11–14]. The plantation system was introduced by the British governors around the mid of the nineteenth century with tea and rubber. They brought Tamil community from South India to Sri Lanka for the purpose of working as laborers in the plantation sector and they now comprise about 5 % of the total population in Sri Lanka [15]. One of the major features of the plantation sector is laborers residing and working in the same plantations for generations. Therefore, Tamil community in the plantation sector has been isolated socio-economically and geographically from the other parts of Sri Lanka. In the twenty-first century, plantation community showed the lowest educational levels, health and nutrition conditions and the highest mortality levels in Sri Lanka [16].

The demographic and Health Survey (DHS) in 2006 reported that 30% of children less than 5 years in the plantation sector of Sri Lanka were underweight (< −2SD weight-for-age), 42% were stunted (< −2SD height-for-age) and 13% were wasted (< −2SD BMI-for-age) [17]. The Literacy rate in the plantation sector of Sri Lanka was 86.1%, while the national average was 95.7% [17]. Infant mortality and low birth weight (less than 2.5 kg) in the plantation sector were 29% and 31%, respectively, while the rates of Urban and Rural sectors were less than 20% [17]. Access to safe water in the plantation sector is 52.4% and the corresponding percentages for Urban and Rural sectors were 98.9% and 90.9% respectively [18]. Nearly 73% of households in the plantation sector had latrine facilities while the national average was 86.7% in 2012 [18]. It is important to determine the true extent and underlying causes of child nutritional status in the plantation sector of Sri Lanka to commence proper intervention strategies to decrease the levels of malnutrition in childhood. However, limited data are available on nutritional status of children and its association with socio-economic characteristics in the plantation sector. Therefore, this study was designed to determine socio-economic characteristics associated with the nutritional status of children in the plantation sector, Sri Lanka.

## Methods

### Study setting and design

A community based cross-sectional study was conducted in three villages surrounded by tea plantations located at Gangawata Korale, Patha Hewaheta and Galaha divisional secretariat divisions in Sri Lanka from January to August 2014. Geographically, these study sites are mountainous areas at the jungle fringes and the mean annual rainfall and temperature ranges between 2000 and 2500 mm and 15 °C to 27 °C respectively. The majority of the inhabitants were employed in tea plantations and factories as unskilled laborers. Although 60% of houses had poured flush toilets, defecation is often done in nearby jungles and no proper water supplements methods were observed in these study areas. Therefore, unprotected water streams close to the residents were used as water sources for their domestic requirements. Generally the education level of the adult population was very low and the majority of people were living in small houses known as line rooms, constructed by the colonial planters about one hundred years ago. The line rooms have small sq. feet with little ventilation, and overcrowding due to the large number of children and their dependent parents.

Children aged 1 to 15 were included in this study and the sample size was calculated using a formula of N = Z^2^Pd/d^2^ with 5% margin of error, 95% confidence interval, and 40% malnourished children in the plantation sector [15]. The calculated minimum sample size was 369. The design effect was taken as 1.5 due to the cluster sampling method was used in this study. Thus the final sample size was 553. A List of names of all children aged between 1 to 15 years in selected tea plantation areas was made with the help of regional administrative and health officers and 553 children were selected randomly using a lottery method for the study. Selected children were categorized into three groups as preschool (1–5 years), children attending to primary schools (6–10 years) and secondary schools (11–15 years) according to their ages and educational status. Children who did not return consent forms, not resident in a study area at least three consecutive years and those suffering from chronic diseases such as heart diseases, kidney and liver failures were excluded from the study.

### Collection of data

Members of the research group visited each selected child and parents or adults in their households and the objectives of the study were explained briefly before the beginning of the study. Written consents were then obtained from parents or guardians of each selected child. Then each parent/guardians who signed the informed written consent were interviewed by members of the research group to elicit information on the educational and employment status of parents, sanitary and household conditions (i.e., type of drinking water, latrine system, number of rooms and housing constructions) and demographic data (i.e., age, gender. Number of siblings and family members) using an interviewer administered structured questionnaire (Additional file 1). Ages of children were obtained from their birth certificates. The questionnaire was prepared in both Sinhala and Tamil languages in order to make the questions clear to the participants.

### Assessment of nutritional status in children

Anthropometric measurements were collected to assess the nutritional status of children. Body weight and height were obtained from each participant at the household level. All children stood barefooted against a vertical wall and height was obtained using a stediometer to the nearest 0.1 cm. Body weight of children with lightweight clothes was measured to the nearest 0.1 kg using a digital balance which was validated before starting the measurement of weight. All measurements were taken by two different research members to minimize the intra personal errors and calculated the mean of these measurements to evaluate final anthropometrical values. Body weight and height were standardized into age specific Z - scores for height-for-age (HAZ), weight-for-age (WAZ) and BMI-for-age (BAZ) by WHO Anthro Plus 1.0.4 and Epi Info 3.5.1 software. Children with scores of WAZ, HAZ and BAZ below than - 2.00 were defined as underweight, stunted and wasted respectively, and more than 2.00 WAZ and BAZ were defined as overweight and obese respectively [19].

### Statistical analysis

Data were entered into Microsoft Excel 2007 and verified with the questionnaire. Data were grouped into three categories: (1) individual characteristics, (2) education and employment status of parents and (3) household characteristics and analyzed statistically using SPSS version 20 (IBM. Somers, NY). Confidence intervals were used in descriptive statistics to describe socio-economic characteristics of the study subjects. Inter observer error of height and weight measurements were determined using a reliability test. The chi-square test was used to analyze the proportions of categorical nutritional indicators (underweight, stunting, wasting, overweight and obese).

Anthropometric measurements of WAZ, HAZ and BAZ were expressed as mean and standard deviations and the variance between three groups (preschool, primary school and secondary school) were analyzed by one-way ANOVA method. Then pairwise comparison of these categories was analyzed by post-hoc Tukey test to determine categories which significantly differed from each other. Multivariate logistic regression using forward elimination model was applied to determine the strength of association of socio-economic variables with under nutritional indicators (stunting, underweight and wasting). For these applications, nutritional indicators were considered as dependant variables while the socio-economic factors were considered as independent variables. Odds ratios with 95% confidence interval were calculated for each factor in logistic regression model. The statistical significant difference was declared if *p* value was less than 0.05.

## Results

### Demographic and socio-economic characteristics

A total of 547 children with a mean age of 7.0 (SD = 3.6) years were participated and 289 (52.8%) of them were male. Out of total participants, 206 (37.7%) were preschool children, 180 (32.9%) were primary school children and 161 (29.4%) were secondary school children. Table 1 shows their demographic, household and parent’s education and employment status of the study subjects. In preschool children, 53% of study subjects were females and 47% were males. In primary and secondary school children, 50% and 55% were females respectively. The number of siblings of study children had ranged from 0 to 6 with the average of 2.6 ± 0.7 and the mean number of family members was 6.8 ± 2.2 (Range 2–12). Overall, the level of education and monthly income of the majority of their parents were very low. However, most of the fathers were employed while the majority of mothers were in homes as housewives. More than 75% children were living in small attached houses made by cement floors, walls and metal laminate roofs. When considering sanitary conditions, more than half of children had latrine facilities. However, most of them had no proper treated drinking water sources.

**Table 1:** Descriptive characteristics of children in the study group

Variables	Preschool children (*n* = 206)		Primary school children (*n* = 180)		Secondary school children (*n* = 161)	
	Mean	95% CI	Mean	95% CI	Mean	95% CI
Individual Characteristics						
Gender (1 = Female, 0 = Male)	53%	(47% - 60%)	50%	(43% - 57%)	55%	(48% - 63%)
No. of Siblings (1 = > 2, 0 = ≤ 2)	30%	(24% - 36%)	28%	(21% - 34%)	24%	(18% - 31%)
Birth Order (1 = > 2, 0 = ≤ 2)	26%	(20% - 32%)	17%	(11% - 22%)	15%	(9% - 20%)
No. of Family members (1 = > 5, 0 = ≤ 5)	61%	(54% - 67%)	57%	(49% - 64%)	53%	(46% - 61%)
Education and Employment status of parents						
Father completed secondary education or above (1 = Yes, 0 = No)	27%	(21% - 33%)	41%	(34% - 49%)	42%	(35% - 50%)
Mother completed secondary education or above (1 = Yes, 0 = No)	41%	(34% - 48%)	41%	(33% - 48%)	32%	(25% - 40%)
Father is employed (1 = Yes, 0 = No)	91%	(87% - 95%)	88%	(83% - 93%)	96%	(93% - 99%)
Mother is employed (1 = Yes, 0 = No)	34%	(28% - 41%)	39%	(32% - 46%)	36%	(29% - 44%)
Household Characteristics						
Type of Dwellings (1 = Attached house, 0 = Separate house)	79%	(74% - 85%)	79%	(73% - 85%)	75%	(68% - 82%)
No. of Rooms (1 = ≤ 2, 0 = > 2)	55%	(48% - 62%)	54%	(47% - 61%)	46%	(38% - 54%)
Floor (1 = Cement, 0 = Earthen)	86%	(81% - 91%)	86%	(80% - 91%)	81%	(75% - 87%)
Wall (1 = Cement, 0 = Wood/mud)	80%	(74% - 85%)	86%	(80% - 91%)	83%	(77% - 89%)
Roof (1 = Metal laminate, 0 = Board laminate)	85%	(81% - 90%)	88%	(83% - 93%)	88%	(83% - 93%)
House own latrine (1 = Yes, 0 = No)	66%	(59% - 72%)	56%	(48% - 63%)	58%	(50% - 65%)
Source of drinking water (1 = Treated, 0 = Untreated)	66%	(59% - 72%)	69%	(62% - 76%)	73%	(66% - 80%)
Monthly income (1 = ≤ 20,000, 0 = > 20,000, Rupees)	77%	(71% - 83%)	73%	(66% - 79%)	77%	(70% - 84%)

### Determinants of child nutritional status

**Fig. 1:**
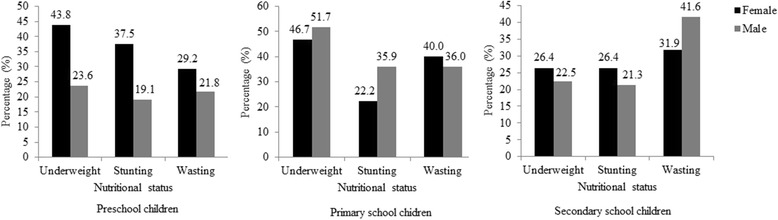
Venn diagrams representing of undernutrition of study subjects

**Fig. 2:**
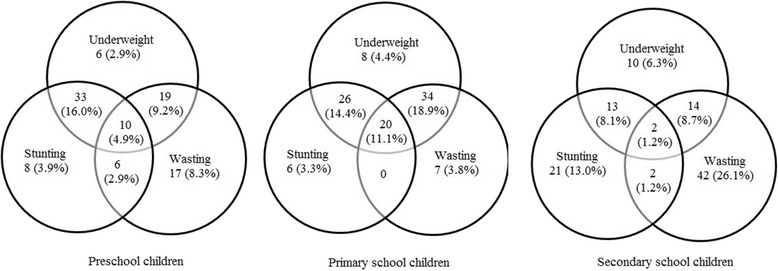
Prevalence of undernutrition by age categories

The reliability coefficient of height was 0.958 and weight was 0. 972. Nutritional status of the study subjects is presented in Table 2. Undernutrition was more common among these children. Overall, high number of children were observed with underweight (*n* = 195, 35.6%), stunting (*n* = 147, 26.9%) and wasting (*n* = 180, 32.9%). However, small numbers of children showed overweight (*n* = 15, 2.7%) and obese (*n* = 17, 3.1%). Of the undernourished children, 53%, 51% and 48% of underweight, stunted and wasting children were males, respectively. More than half of undernourished children lived with large number of family members and most of their parents’ had low educational background. Monthly family income of many undernourished (> 70%) children was less than 20,000 Sri Lankan rupees (133 USD) and lived in small attached houses consisting one or two rooms. In addition, children had not house own latrine facilities showed high prevalence of undernutrition compare to children lived in houses which had latrine facilities (Table 2). When considering the preschool children, undernutrition comparatively more common in males than females. In contrast, the level of undernutrition among school female children was higher than male children. Figure 1 shows that secondary school children had the highest percentage of one type of undernutrition conditions (Underweight or stunting or wasting) while two or more than two types of undernutrition conditions were more common among primary school children. Underweight was the commonest undernutrition condition among preschool and primary school children while wasting was the commonest in secondary school children (Fig. 2). With respect to children’s groups, undernutrion was more common in primary school children while secondary school children showed the highest proportions of overnutrition conditions comparable to other groups. Underweight, wasting and all Z score nutritional indicators were significantly associated with these children’s groups (Table 3). Primary school children showed lowest HAZ and BAZ mean Z score while the lowest mean Z score for WAZ remained among secondary school children. According to Tukey test, mean WAZ and mean BAZ in primary school children were significantly lower from the other two groups (WAZ: Pre and primary school child *p* < 0.001, secondary and primary school children *p* = 0.033; BAZ: Pre and primary school children *p* = 0.001, secondary and primary school children *p* = 0.035) and Mean HAZ was significantly lower than preschool children (*p* = 0.005).

**Table 2:** Demographic and socioeconomic characteristics with nutritional status in children

Variables	WAZ		HAZ		BAZ	
	Normal	Underweight	Normal	Stunting	Normal	Wasting
	(*N* = 352)	(*n* = 195)	(*n* = 400)	(*n* = 147)	(*n* = 367)	(*n* = 180)
Individual Characteristics						
Gender (1 = Female, 0 = Male)	197 (56.0%)	92 (47.8%)	217 (54.2%)	72 (49.0%)	196 (53.4%)	93 (51.7%)
No. of Siblings (1 = > 2, 0 = ≤ 2)	96 (27.3%)	55 (28.2%)	112 (28.0%)	39 (26.5%)	98 (26.7%)	53 (29.4%)
Birth Order (1 = > 2, 0 = ≤ 2)	71 (20.2%)	36 (18.5%)	75 (18.8%)	32 (21.8%)	78 (21.3%)	29 (16.1%)
No. of Family members (1 = > 5, 0 = ≤ 5)	200 (56.8%)	113 (57.9%)	233 (58.3%)	80 (54.7%)	210 (57.2%)	103 (57.2%)
Education and Employment status of parents						
Father completed secondary education or above (1 = Yes, 0 = No)	135 (38.4%)	65 (33.3%)	144 (36.0%)	56 (38.1%)	139 (37.9%)	61 (33.9%)
Mother completed secondary education or above (1 = Yes, 0 = No)	135 (38.4%)	75 (38.5%)	146 (36.5%)	64 (43.5%)	155 (42.2%)	55 (30.6%)
Father is employed (1 = Yes, 0 = No)	322 (91.5%)	179 (91.8%)	367 (91.8%)	134 (91.2%)	338 (92.1%)	163 (90.6%)
Mother is employed (1 = Yes, 0 = No)	141 (40.1%)	58 (29.7%)	142 (35.5%)	57 (38.8%)	148 (40.3%)	51 (28.3%)
Household Characteristics						
Type of Dwellings (1 = Attached house, 0 = Separate house)	276 (78.4%)	151 (77.4%)	318 (79.5%)	109 (74.1%)	283 (77.1%)	144 (80.0%)
No. of Rooms (1 = ≤ 2, 0 = > 2)	182 (51.7%)	103 (52.8%)	200 (50.0%)	85 (57.8%)	197 (53.7%)	88 (48.9%)
Floor (1 = Cement, 0 = Earthen)	301 (85.5%)	160 (82.1%)	343 (85.8%)	118 (80.3%)	305 (83.1%)	156 (86.7%)
Wall (1 = Cement, 0 = Wood/mud)	293 (83.2%)	159 (81.5%)	333 (83.3%)	119 (81.0%)	302 (82.3%)	150 (83.3%)
Roof (1 = Metal laminate, 0 = Board laminate)	302 (85.8%)	174 (89.2%)	348 (87.0%)	128 (87.1%)	316 (86.1%)	160 (88.9%)
House own latrine (1 = Yes, 0 = No)	205 (58.2%)	123 (63.1%)	244 (61.0%)	84 (57.1%)	223 (60.8%)	105 (58.3%)
Source of drinking water (1 = Treated, 0 = Untreated)	114 (32.4%)	57 (29.2%)	127 (31.8%)	44 (29.9%)	116 (31.6%)	55 (30.6%)
Monthly income (1 = ≤ 20,000, 0 = > 20,000 Rupees)	268 (76.1%)	146 (74.9%)	302 (75.5%)	112 (76.2%)	269 (77.3%)	145 (80.6%)

**Table 3:** Nutritional characteristics of the study sample

Nutritional status	Pre school	Primary school	Secondary school	*p* value
	Children (*n* = 206)	children (*n* = 180)	children (*n* = 161)	
Underweight (< −2SD WAZ)	68 (33.0%)	88 (48.9%)	39 (24.2%)	< 0.001*
Stunted (< −2SD HAZ)	57 (27.7%)	52 (28.9%)	38 (23.6%)	0.518
Wasted (< −2SD BAZ)	52 (25.2%)	68 (37.8%)	60 (37.3%)	0.012*
Overweight (> 2SD WAZ)	3 (1.5%)	5 (2.8%)	7 (4.3%)	0.242
Obese (> 2SD BAZ)	5 (2.4%)	5 (2.8%)	7 (4.3%)	0.548
Mean WAZ score	−1.54 (1.15)	−2.01 (0.96)	−1.76 (0.34)	< 0.001*
Mean HAZ score	−1.05 (1.53)	−1.42 (0.92)	−1.29 (0.78)	0.006*
Mean BAZ score	−1.30 (1.17)	−1.67 (1.01)	−1.96 (0.87)	< 0.001*

*statistically significant association

### Risk factors of child nutritional status

The risk factors associated with nutritional status in relation to socio-economic characteristics among these communities were analyzed by multivariate logistic regression method. Table 4 shows that female children were 2.9 times more likely to be underweight (OR = 2.89; CI = 1.50–5.55) and 2.8 times more likely to be stunted (OR = 2.75; CI = 1.39–5.45) in preschool children. Wasting was significantly correlated with birth order (OR = 6.39; CI = 1.23–33.08), number of siblings (OR = 3.16; CI = 1.11–8.97) and the employment of mothers were (OR = 2.12; CI = 1.05–4.28). In primary school children, family members were 2.3 times (OR = 2.31; CI = 1.13–4.74) and one or two room houses were 2.2 times (OR = 2.15; CI = 1.11–4.15) more likely to be underweight. Similarly, children had occupied and educated mothers were 2 times (OR = 2.03; CI = 1.02–3.63) and 4.1 times (OR = 4.09; CI = 1.86–9.00) more likely to be underweight and wasting respectively (Table 5).

**Table 4:** Multivariate analysis of risk factors for nutritional status of preschool children

Variables	Underweight		Stunting		Wasting	
	OR (95% CI)	*p* value	OR (95% CI)	*p* value	OR (95% CI)	*p* value
Individual Characteristics						
Female	2.89 (1.50–5.55)	0.001*	2.75 (1.39–5.45)	0.004*	1.58 (0.78–3.21)	0.207
More than 2 siblings	1.50 (0.57–3.93)	0.412	1.39 (0.51–3.79)	0.517	3.16 (1.11–8.97)	0.031*
More than 2 birth Order	3.11 (0.73–13.35)	0.127	2.23 (0.50–10.19)	0.303	6.39 (1.23–33.08)	0.027*
More than five family members	1.03 (0.53–2.01)	0.938	1.08 (0.54–2.17)	0.832	1.60 (0.79–3.26)	0.195
Education and Employment status of parents						
Father completed secondary education or above	0.42 (0.18–0.97)	0.041	0.54 (0.24–1.25)	0.153	0.75 (0.32–1.75)	0.501
Mother completed secondary education or above	0.53 (0.27–1.08)	0.079	0.71 (0.33–1.51)	0.374	0.62 (0.30–1.24)	0.175
Employed father	1.96 (0.51–6.51)	0.324	5.61 (0.96–31.08)	0.058	0.45 (0.13–1.59)	0.215
Employed mother	2.12 (1.05–4.28)	0.037*	1.40 (0.68–2.86)	0.361	2.12 (1.05–4.28)	0.037*
Household Characteristics						
Attached houses	0.76 (0.33–1.73)	0.506	1.16 (0.51–2.63)	0.730	0.69 (0.27–1.55)	0.438
One/two rooms in a household	0.97 (0.52–1.86)	0.900	0.96 (0.82–2.90)	0.064	0.82 (0.41–1.68)	0.590
Cement floor	0.88 (0.35–2.32)	0.791	1.74 (0.62–4.88)	0.293	0.88 (0.32–2.44)	0.800
Cement wall	0.79 (0.35–1.80)	0.576	0.49 (0.21–1.19)	0.118	0.79 (0.32–1.91)	0.593
Metal laminate roof	0.69 (0.22–2.17)	0.522	1.62 (0.59–4.42)	0.348	0.78 (0.28–2.18)	0.634
House own latrine	1.13 (0.57–2.23)	0.740	0.84 (0.41–1.72)	0.639	0.65 (0.31–1.37)	0.262
Drinking treated water	0.94 (0.47–1.87)	0.860	1.69 (0.83–3.42)	0.145	1.07 (0.52–2.28)	0.816
Less than Rs 20,000 monthly income (USD 133)	1.87 (0.83–4.17)	0.128	1.25 (0.55–2.81)	0.594	0.63 (0.68–1.25)	0.066

*OR* odds ratio, *CI* confidence interval

*Statistically significant association

**Table 5:** Multivariate analysis of risk factors for nutritional status of primary school children

Variables	Underweight		Stunting		Wasting	
	OR (95% CI)	*p* value	OR (95% CI)	*p* value	OR (95% CI)	*p* value
Individual Characteristics						
Female	0.71 (0.37–1.34)	0.287	0.45 (0.22–0.93)	0.031	1.25 (0.63–2.47)	0.525
More than two Siblings	1.28 (0.61–2.69)	0.523	1.10 (0.48–2.51)	0.823	0.89 (0.40–1.96)	0.887
More than two birth Order	1.43 (0.39–5.14)	0.590	2.46 (0.59–10.29)	0.216	2.71 (0.67–10.90)	0.160
More than five family members	2.31 (1.13–4.74)	0.022*	1.57 (0.71–3.48)	0.263	0.78 (0.36–1.63)	0.492
Education and Employment status of parents						
Father completed secondary education or above	0.74 (0.38–1.45)	0.380	0.74 (0.36–1.52)	0.403	0.64 (0.32–1.32)	0.228
Mother completed secondary education or above	1.32 (0.64–2.72)	0.448	0.69 (0.32–1.48)	0.341	0.33 (0.15–0.72)	0.005*
Employed father	0.67 (0.23–1.94)	0.457	0.50 (0.17–1.47)	0.207	0.50 (0.15–1.64)	0.253
Employed mother	2.03 (1.02–3.63)	0.045*	1.20 (0.57–2.56)	0.630	4.09 (1.86–9.00)	0.001*
Household Characteristics						
Attached house	1.57 (0.68–3.62)	0.287	2.22 (0.96–5.11)	0.062	0.56 (0.23–1.41)	0.217
One/two Rooms in a household	2.15 (1.11–4.15)	0.023	1.26 (0.62–2.55)	0.517	1.56 (0.79–3.09)	0.200
Cement Floor	1.07 (0.42–2.72)	0.892	1.42 (0.52–3.87)	0.502	0.85 (0.38–2.22)	0.578
Cement Wall	1.96 (0.74–5.21)	0.178	1.30 (0.48–3.54)	0.605	1.99 (0.73–5.39)	0.174
Metal laminate roof	0.59 (0.22–1.66)	0.323	0.86 (0.29–2.53)	0.789	0.41 (0.12–1.29)	0.128
House own latrine	1.94 (0.99–3.81)	0.054	0.79 (0.36–1.52)	0.408	0.72 (0.35–1.41)	0.217
Drinking treated water	1.14 (0.77–1.69)	0.518	1.12 (0.73–1.71)	0.601	1.06 (0.71–1.59)	0.756
Less than Rs 20,000 monthly income (USD 133)	1.90 (0.93–3.88)	0.078	2.51 (1.08–5.82)	0.032*	1.29 (0.61–2.71)	0.500

*OR* odds ratio, *CI* confidence interval

*Statistically significant association

In secondary school children, family members and low monthly income were 2.9 times (OR = 2.93; CI = 1.15–7.41 and OR = 2.90; CI = 1.05–8.06) more likely to be underweight. However, paternal education (OR = 0.33; CI = 0.15–0.76) and employment (OR = 0.37; CI = 0.14–0.99) were predictive factors for wasting (Table 6). Household latrine facilities, drinking treated water and the types of wall, roof and floor were not significantly associated with any group of child nutritional status.

**Table 6:** Multivariate analysis of risk factors for nutritional status of secondary school children

Variables	Underweight		Stunting		Wasting	
	OR (95% CI)	*p* value	OR (95% CI)	*p* value	OR (95% CI)	*p* value
Individual Characteristics						
Female	1.16 (0.46–2.91)	0.752	1.75 (0.67–4.59)	0.256	0.75 (0.32–1.80)	0.525
More than two siblings	2.13 (0.75–6.04)	0.156	1.13 (0.42–3.03)	0.814	1.15 (0.47–2.83)	0.758
More than two birth Orde	0.45 (0.08–2.28)	0.332	1.07 (0.19–6.01)	0.935	3.92 (0.74–5.79)	0.109
More than five family members	2.93 (1.15–7.41)	0.024*	0.75 (0.29–1.98)	0.566	1.93 (0.84–4.43)	0.122
Education and Employment status of parents						
Father completed secondary education or above	0.79 (0.33–1.89)	0.602	2.29 (0.94–5.58)	0.068	0.33 (0.15–0.76)	0.009*
Mother completed secondary education or above	1.65 (0.65–4.18)	0.291	1.26 (0.52–3.05)	0.608	1.65 (0.65–4.18)	0.291
Employed father	0.93 (0.33–2.63)	0.889	1.45 (0.55–3.87)	0.488	0.37 (0.14–0.99)	0.046
Employed mother	0.79 (0.33–7.91)	0.090	1.95 (0.81–4.66)	0.136	1.11 (0.49–2.45)	0.805
Household Characteristics						
Attached house	1.24 (0.43–3.56)	0.689	0.65 (0.21–2.04)	0.462	1.39 (0.50–3.85)	0.531
One/two rooms in a household	0.64 (0.28–1.43)	0.278	0.91 (0.39–2.09)	0.823	1.77 (0.82–3.84)	0.149
Cement Floor	1.95 (0.76–4.98)	0.164	0.37 (0.09–1.47)	0.151	0.44 (0.16–1.21)	0.114
Cement Wall	1.05 (0.37–2.96)	0.931	2.65 (0.97–6.98)	0.055	0.51 (0.18–1.45)	0.206
Metal laminate Roof	0.57 (0.15–2.22)	0.415	1.14 (0.26–4.98)	0.855	1.46 (0.43–4.90)	0.542
House own latrine	1.89 (0.75–4.74)	0.179	1.00 (0.41–2.45)	1.000	1.48 (0.64–3.41)	0.361
Drinking treated water	1.15 (0.46–2.89)	0.764	1.27 (0.50–3.23)	0.613	1.25 (0.53–2.94)	0.604
Less than Rs 20,000 monthly income (USD 133)	2.90 (1.05–8.06)	0.040*	1.32 (0.48–3.64)	0.587	0.72 (0.26–1.88)	0.704

*OR* odds ratio, *CI* confidence interval

*Statistically significant association

## Discussion

Malnutrition causes children more susceptible to infections. Those frequent infections negatively affect their physical and cognitive abilities locking them into a vicious cycle. This study confirms that undernutrition is still a considerable health problem in the plantation sector in Sri Lanka. This is much similar to the prevalence of malnutrition reported by the DHS of Sri Lanka in 2006 [17]. However, National Nutrition and Micronutrient Survey in Sri Lanka reported that 15.1%, 27.3% and 21.9% of children aged between 6 and 59 months had stunting, underweight and wasting respectively in 2012 [20]. It is commonly believed that these differences result from a low standard of living, high levels of infectious diseases, and low purchasing ability, all of which have an effect on feeding practices [21].

In the present study, primary school children are more likely to be undernourished than preschool and secondary school children. Preschool children, mainly depend on breastfeeding and may be protected by the mother’s immune system at birth [22]. Breast milk protects small children from infectious diseases and finally affects their nutritional status. With the increasing of age, children start to take complementary food in addition to breastfeeding and get nutrition from many food sources. Some studies have reported that the deviation of normal growth rates in children starts from several months after birth because breastfeeding is no longer enough to fulfill the requirements of growing children [23]. Inadequate complementary foods also might be a reason of increasing trends of malnutrition in older children. This might be attributed by stopping or reducing breastfeeding and insufficient intake of complementary food.

Underweight was the most common undernutrition indicator among these children. WAZ reflects body mass relative to age and it fluctuates with time and therefore it has a role in acute as well as chronic malnutrition [24]. High prevalence of stunting and wasting also reveal that most of the children in the study groups were suffering from long term chronic malnutrition, which negatively effect on both mental and physical improvements in childhood. Decreasing food purchasing ability, dietary changes and poor personal hygiene of mothers as well as infectious diseases may have a role in the nutritional status of children [21]. The present study revealed that primary school children showed the lowest mean Z scores of WAZ and HAZ compare to preschool and secondary school children. Since undernutrition increases with age, it reflects longer exposure to chronic malnutrition is common among children in this community. Knowledge of food nutrients and improved hygiene are a few of the factors that could be involved in reducing the prevalence of undernutrition among secondary school children.

Social customs and norms in communities and families describe the roles of members in households. In Asia, a number of the restriction process consists regarding the health of small children [25]. This study showed that preschool female children had significantly higher rates of underweight and stunting than male in this community. This is inconsistent with the studies in India and Bangladesh reported that moderate and severe undernutrition was higher in female [26, 27]. Gender inequality of male preference over female in care and feeding practices could be a major reason for this finding and it is commonly seen in many cultures and social beliefs in Asia [28]. In addition, Religious influences also discourage the consumption of some healthy foods. Therefore, child feeding practices of mothers and caregivers may affect dietary intake of children in this community. This indicates that more attention on the maternal characteristics at the household level should be paid for the improvement of child health particularly in preschool stage.

Education generates knowledge and favorable environment that positively affects for child health. In the present study, a declined trend of child malnutrition is observed where the educational level of mothers increased. Similar findings were reported in numerous studies showing children with low educated mothers had higher prevalence of undernutrition than highly educated mothers [29–32]. Education makes the mothers inform nutritional values of food and get more understandings of child physical and mental growth. Underage marriages are still common in the plantation sector and most of young low educated, married girls are neither mentally nor physically ready for motherhood. They often have a greater chance of giving low birth weights children [33]. This could be another factor for child undernutrition in the plantation sector. National Family Health Survey in India revealed that underage marriage mothers were a contributing factor for undernutrition of their children [34].

The main source of income for most families in these communities is working as laborers in tea plantations on a day-to-day basis. Because of the uncertainty in these occupations, both parents prefer to work in the industrial sector or migrate to Middle Eastern countries. Therefore, parents, particularly mothers have very limited time to prepare meals and feed children at home. Most of the children in the plantation sector spend their day time with grandparents or other relatives and this could be disadvantaged on children’s physical and mental growth and development particularly in preschool and primary school children [33]. Children of unemployed mothers were better nourished than the children whose mothers were employed [35, 36]. In addition, employment of mothers outside of their homes creates many social problems in family level and it negatively affect for the physical and mental development of children [37]. In many cultures in Asia, father is the money earner and the decision maker at the household level. Highly educated fathers more care of their children and provide health facilities when they need. However, in this study, high educated and occupied fathers play a significant role in decreasing the rate of wasting among secondary school children. This could be an ability to make decisions about getting good quality food for children [38, 39]. Further studies are needed to determine how parents’ educational influences to the nutritional status of children.

Generally the majority of residents in the plantation sector live in the same places where their previous generations were lodged called “Line houses” without major modifications. In this community, 68% children lived in long terraces of one or two room houses attached to each other under minimal socio-economic conditions. Further, few subjects were living in quarters and detached houses. The high prevalence rate of underweight among children in attached houses indicates that poverty and poor sanitary conditions were common in this study group. Similarly, a survey in India found that the prevalence of undernutrition among children in low standard households increases two times than the children living in high standard households [40]. In the present study, we revealed that sanitary and treated water facilities were inadequate in this community. This causes the high prevalence of repeated infectious diseases still remain in the plantation sector [11–13, 41–43]. Inadequate dietary intake weakens the responses of the immune system in children and increases vulnerability to infections. Several studies have been confirmed that infections were commonly diagnosed in children suffering from undernutrition [44, 45]. Another study identified that soil of the plantation sector of Sri Lanka has been contaminated with helminth eggs [46]. When handling contaminated soil, water or food those infective stages of micro-organisms can enter into the body and initiate diseases. This association between undernutrition and infection generates a cycle of repeated illness and deteriorating nutritional status. Critical nutrition specific interventions include promoting micronutrient supplementation, breastfeeding practices, access to clean drinking water and good sanitation practices and reducing the incidence of underage marriage of girls and delivering children with low birth weights can break this cycle and enhance their nutrition status [47, 48]. Although, hospitals and dispensaries in the plantation sector provide basic medical services to the estate inhabitants, lack of qualified health staffs and medical facilities are the major difficulties faced by the health service in the plantation sector [49].

In the present study, majority of undernourished children has more than five family members. Poor allocation of household resources cannot fulfill the nutritional requirements of each member equally. Particularly, children in large family size have poor access to sufficient and quality food [50]. This is consistent with several studies in India that have identified that the large family size negatively affect for child nutritional status [51, 52]. In addition, the presence of many siblings is also a risk factor for undrenutrition due to limited availability of time for care and feeding of each child [53]. Another study has reported that the food availability of children in large families were lower than the children in small families [54]. Birth order affects the attention of mothers to intra household activities and child care. In the present study, higher birth order has a negative impact on wasting in preschool children. This reflects the nutritional status of the first children to be better than that of last children. However, in the wide confidence interval (CI = 1.23–33.08) means that the data on wasting are too inconstant to make a reliable estimate. Families with more children have a high economic burden for the consumption of food and thus majority of young children are likely to be undernutrition [47]. Similarly, children in higher birth order (>3) were prone to undernutrition than those with low birth order in India [55]. Family planning programs promoting the reduction of the size and increasing the gap between childbirths will reduce the numbers of malnourished children from higher birth rates.

More than half of children use common latrine facilities in this community and shows a higher prevalence of underweight than those used household latrines. This might be explained by the poverty and lack of good quality latrines. Similarly, several studies have revealed a large number (>50%) of children living in tea plantations in Sri Lanka used poorly defined public toilets and open ground for their defecation [11–13]. Water purification centers were not available in this area and water from unprotected storage tanks were used for domestic requirements (i.e., drinking and washing clothes). Studies conducted in Sudan and Philippines revealed that improved water and better quality sanitation facilities positively affect, for good health conditions of children [56, 57]. Some studies have been reported that untreated water causes diarrheal diseases and positively correlated with the malnutrition of children [58, 59]. Therefore, we suggest that sanitary facilities affect the nutritional growth irrespectively of social demographic characteristics.

High household income of the family offers the opportunity to provide good quality food and more health services. Another study shows the links paternal education is a strong determinant for enhancing the household income in developing countries [60]. Household Income and Expenditure Survey in 2012/2013 reported that mean income per household in the estate sector were 30,220 (USD 201) Sri Lankan Rupees (SLR) while the national level was SLR 45878 (USD 305). However the majority of this community had less than 20,000 rupees (USD 133) per month income [18]. In this study, Underweight was more common in lower income groups than children in higher income households. This is similar to the studies conducted in India showed that children in low income groups had a high rate of undrenutrition [61]. This indicates that poverty is not the only factor responsible for undernutrition but inadequate dietary intake, infection, poor hygienic habits and the environment and low education status might contribute to child undernutrition among low income groups. Access to higher education for women and family planning programs, improvement of sanitary facilities and the socioeconomic conditions are major factors to enhance the child nutritional status of this community. However, an obvious limitation is this study was not powered for many of the comparison made and it was only powered to estimate an overall prevalence for all children 1–15 years old.

## Conclusions

Parents’ education and occupation, birth order, number of siblings, family income and gender are main attributes to the undernutrition in this community. Government as well as non-government organizations need to prioritize to implement community based programs to enhance the knowledge of child health and decrease the level of undernutrition. In addition, future detailed studies are required to determine factors affecting the nutritional status of children in this study area.

## Additional file

Additional file 1: Questionnaire of the study. Nutritional status and correlated socioeconomic factors among preschool and school children in plantation communities, Sri Lanka - Questionnaire. Questions asked from participants to get their socioeconomic characteristics. (DOCX 19 kb)

## Abbreviations

BAZ: Body mass index-for-age
CI: Confidence interval
DHS: Demographic and Health Survey
HAZ: Height-for-age
OR: Odds ratio
WAZ: Weight-for-age
WHO: World health organization

## References

[1] Schroeder DG, Brown KH (1994). Nutritional status as a predictor of child survival: summarizing the association and quantifying its global impact. Bull WHO.

[2] Pelletier DL, Frongillo EA, Schroeder DG, Habicht JP (1994). A methodology for estimating the contribution of malnutrition to child mortality in developing countries. J Nutr.

[3] Pelletier DL. Malnutrition, morbidity and child mortality in developing countries. In Too young to die: Genes or Gender? Edited by: United Nations. New York: Department of Economic and Social Affairs, Population Division; 1998:109–32.

[4] Pelletier DL, Frongillo EA, Habicht JP (1993). Epidemiologic evidence or a potentiating effect of malnutrition on child mortality. Am J Pub Health.

[5] Ubesie AC, Ibeziakor NS (2012). High burden of protein-energy malnutrition in Nigeria: beyond the health care setting. Ann Med Health Sci Res.

[6] Jesmin A, Yamamoto SS, Malik A, Haque A (2011). Prevalence and determinants of chronic malnutrition among preschool children. J Health Popul Nutr.

[7] Dewey KG, Begum KB (2011). Long-term consequences of stunting in early life. Maternal and Child Nutr.

[8] Gluckman PD, Hanson MA (2004). Living with the past: evolution, development, and patterns of disease. Science.

[9] Abuya BA, Ciera JM, Kimani-Murage E (2012). Effect of mother’s education on child’s nutritional status in the slums of Nairobi. BMC Pediatr.

[10] Asfaw M, Wondaferash M, Taha M, Dube L (2015). Prevalence of undernutrition and associated factors among children aged between six to fifty nine months in Bule Hora district. South Ethiopia. BMC Pub Health..

[11] De Siva NR, De Silva HJ, Jayapani VP (1993). Intestinal parasitoses in the Kandy area Sri Lanka. Southeast Asian J Trop Med Public Health.

[12] Gunawardena GSA, Karunaweera ND, Ismail MM (2004). Socio- economic behavioral factor affecting the prevalence of Ascaris infection in a low-country tea plantation in Sri Lanka. Ann Trop Med Parasit.

[13] Sorensen E, Ismail M, Amarasinghe DK, Hettiarachchi I, Dassenaike TS (1996). The prevalence and control of soil-transmitted nematode infections among children and women in the plantations in Sri Lanka. Ceylon Med J..

[14] Gunawardena K, Kumarendran B, Ebenezer R, Gunasinghe MS, Pathmeswaran A, De Silva N (2011). Soil-tranmitted helminth infections among plantation sector school children in Sri Lanka prevalence after ten years of preventive chemotherapy. PLoS Negl Trop Dis.

[15] Ahmed HI (2014). Estate Tamils of Sri Lanka – a socio economic review. Int J Soc Anthropol.

[16] Kurihara S (2011). Tea estate plantation community in NuwaraEliya District of Sri Lanka: an introductory overview of social issues and poverty among residents living under the conventional plantation system. Yokohama J Soc Sci.

[17] Department of Census and Statistics in collaboration with Ministry of Health, Sri Lanka Demographic and Health Survey 2006/2007. Colombo, Sri Lanka; 2007.

[18] Household Income and Expenditure Survey - 2012/13. Department of Census and Statistics Ministry of policy planning economic Affairs, child youth and cultural Affairs, Colombo Sri Lanka; 2015.

[19] World Health Organization. Physical status: The use and interpretation of anthropometry. Geneva: World Health Organization. 1995. 8594834

[20] Ministry of Health, Sri Lanka and UNICEF. National nutrition and micro nutrient survey 2012: Anaemia among children aged 6–59 months and nutritional status of children and adults. Ministry of Health, Sri Lanka. http://www.unicef.org/srilanka/MNS Report. 2013.

[21] Bain LE, Awah PK, Geraldine N, Kindong NP, Sigal Y, Bernard N, Tanjeko AT (2013). Malnutrition in sub – Saharan Africa: burden, causes and prospects. Pan Afr Med J.

[22] Kandala NB, Fahrmeir L, Klasen S, Priebe J (2009). Geo-additive models of childhood Undernutrition in three sub-Saharan African countries. Population, Space and Place.

[23] Lewis IA (1999). Young child feeding practices in Nigeria in complementary feeding of young children in Africa and the Middle East.

[24] Lindsay P. Nutritional disorders. Care of the Adult with Intellectual Disability in Primary Care. iffe publishing. London: 2011; plRadc147.

[25] Zaidi AKM, Awasthi S, De Silva HJ (2004). Burden of infectious disease in South Asia. BMJ.

[26] Banerjee B, Mandal ON (2005). An intervention study in malnutrition among infants in a tribal community of West Bengal. Indian J Community Med.

[27] Khan MM, Kraemer A (2009). Factors associated with being underweight, overweight and obese among ever-married non-pregnant urban women in Bangladesh. Singap Med J.

[28] Klasen S (2008). Poverty, undernutrition, and child mortality: some interregional puzzles and their implications for research and policy. J Econ Inequality.

[29] Frost MB, Forste R, Haas DW (2005). Maternal education and child nutritional status in Bolivia: finding the links. Soc Sci Med.

[30] Handa S (1999). Maternal education and child height. Econ Devel Cult Change.

[31] Abuya BA, Onsomu EO, Kimani JK, Moore D (2011). Influence of maternal education on child immunization and stunting in Kenya. Matern Child Health J.

[32] Duru CB, Oluoha UR, Uwakwe KA, Diwe KC, Merenu IA, Chigozie IO, Iwu AC (2015). Prevalence and socio demographic determinants of malnutrition among under-five children in rural communities in Imo state. Nigeria Am J Pub Health Res.

[33] Townsend N, Williams J, Wickramasinghe K, Karunarathne W, Olupeliyawa A, Manoharan S, Friel S. Barriers to healthy dietary choice amongst students in Sri Lanka as perceived by school principals and staff. Health Promotion Int. 2015:1–11. 10.1093/heapro/dav056 28180258

[34] Corsi DJ, Mejía-Guevara I, Subramanian SV (2016). Risk factors for chronic undernutrition among children in India: estimating relative importance, population attributable risk and fractions. Soc Sci Med.

[35] Powell CA, Grantham-McGregor S (1985). The ecology of the nutritional status and the development in young children in Kingston, Jamaica. Am J Clin Nutr.

[36] Yeleswarapu BK, Nallapu SSR (2012). A. Comparative study on the nutritional status of the pre-school children of the employed women and the unemployed women in the urban slums of Guntur. J Clin Diagn Res.

[37] Islam MA1, Rahman MM, Mahalanabis D. Maternal and socioeconomic factors and the risk of severe malnutrition in a child: a case-control study. Eur J Clin Nutr. 1994;48:416–424. 7925224

[38] Amsalu S, Tigabu Z (2008). Risk factors for severe acute malnutrition in children under the age of five. Ethiop J Health Dev.

[39] Islam MM, Alam M, Tariquzaman M, Kabir MA, Pervin R, Begum M (2013). Predictors of the number of under-five malnourished children in Bangladesh: application of the generalized Poisson regression model. BMC Public Health.

[40] National Family Health Survey (NFHS) II Report (1998-1999) Ministry of Health and Family Welfare India 2010. http://rchiips.org/nfhs/pub_nfhs-2.shtml. Accessed 01 Oct 2010.

[41] Galgamuwa GLS, Iddawela WMDR, Dharmaratne SD (2015). Development and piloting of Ascariasis surveillance system of children in Sri Lanka. Online Journal of Public Health Informatics.

[42] Galgamuwa LS, Iddawela WMDR, Dharmaratne SD (2016). Intestinal protozoa infections, associated risk factors and clinical features among children in a low-income tea plantation community in Sri Lanka. Int J Community Med Public Health.

[43] Suraweera OSA, Galgamuwa LS, Iddawela D, Wickramasinghe S (2015). Prevalence and associated factors of *Enterobius vermicularis* infection in children from a poor urban community in Sri Lanka: a cross-sectional study. Int J Res Med Sci.

[44] Umamaheswari B, Biswal N, Adhisivam B, Parija SC, Srinivasan S (2010). Persistent diarrhea: risk factors and outcome. Indian J Pediatr.

[45] Sharma ML. A study of malnutrition and associated infection in children in an urban private hospital in India. Malnutrition and Infection. 2001; http://www.capgan.org/malinf.htm. Accessed 03 Oct 2010

[46] Edirisinghe JS, Weligama DJ. Soil contamination with geohelminth ova in a tea plantation. Ceylon Med J 1997;42:167–72. 9476399

[47] Asfaw M, Wondaferash M, Taha M, Dube L (2015). Prevalence of undernutrition and associated factors among children aged between six to fifty nine months in Bule Hora district. South Ethiopia BMC Pub Health.

[48] Improving child nutrition. The achievable imperative for global progress United Nations Children’s Fund (UNICEF). New York, USA.2013.

[49] Gwatkin D, Rustein S, Kierston J, Suliman E, Wagstaff A, Agbessi A (2005). Socioeconomic differences in health, nutrition and population.

[50] Fentaw R, Bogale A, Abebaw D (2013). Prevalence of child malnutrition in agro-pastoral households in afar regional state of Ethiopia. Nutr Res Pract.

[51] Saloojee H, De Maayer T, Garenne ML, Kahn K (2007). What's new? Investigating risk factors for severe childhood malnutrition in a high HIV prevalence South African setting. Scand J Public Health.

[52] Lima Mde C, Motta ME, Santos EC (2004). Pontes da Silva GA. Determinants of impaired growth among hospitalized children: a case-control study Sao Paulo Med J.

[53] Basit A, Nair S, Chakraborthy K, Darshan B, Kamath A (2012). Risk factors for under-nutrition among children aged one to five years in Udupitaluk of Karnataka, India: a case control study. Australas Med J.

[54] Ajao KO, Ojofeitimi EO, Adebayo AA, Fatusi AO, Afolabi OT (2010). Influence of family size, household food security status, and child care practices on the nutritional status of under-five children in Ile-Ife. Nigeria Afr J Reprod Health.

[55] Harishankar, Dwivedi S, Dabral SB, Walia DK. Nutritional status of children under 6 years of age. Indian J Prev Soc Med. 2004;35:156–62.

[56] Merchant AT, Jones C, Kiure A, Kupka R, Fitzmaurice G, Herera MG, Fowsi WW (2003). Water and sanitation associated with improved child growth. Eur J Clin Nutri.

[57] Magnani RJ, Mock NB, Bertrand WE, Clay DC (1993). Breastfeeding, water and sanitation, and childhood malnutrition in the Philippines. J Bio soc Sci.

[58] Alam DS, Marks GC, Baqui AH, Yunus M, Fuchs GJ (2000). Association between clinical type of diarrhea and growth of children under 5 years in rural Bangladesh. Int J Epidemiol.

[59] Guerrant RL, Schorling JB, McAuliffe JF, de Souza MA (1992). Diarrhea as a cause and an effect of malnutrition: diarrhea prevents catch-up growth and malnutrition increases diarrhea frequency and duration. AmJTrop Med Hyg.

[60] Haddad L, Alderman H, Appleton S, Song L, Yohannes Y (2003). Reducing child malnutrition: how far does income growth take us?. The World Bank Economic Review.

[61] Bhutia DT (2014). Protein energy malnutrition in India: the plight of our under five children. J Family Med Prim Care.

