# Supplementary material for: Nutritional status and correlated socio-economic factors among preschool and school children in plantation communities, Sri Lanka
Source: BMC Public Health. 2017 May 2;17:377. doi: 10.1186/s12889-017-4311-y (PMC5414369; doi:10.1186/s12889-017-4311-y)
Supplement: Additional file 1: — Questionnaire of the study. Nutritional status and correlated socioeconomic factors among preschool and school children in plantation communities, Sri Lanka - Questionnaire. Questions asked from participants to get their socioeconomic characteristics. (DOCX 19 kb) [file 12889_2017_4311_MOESM1_ESM.docx]

**Nutritional status and correlated socioeconomic factors among preschool and school children in plantation communities, Sri Lanka**

**Questionnaire**

Name - …………………………………………………….. Age years

Education level -……………………………………. ……….. Sex M F

1.) Number of members in the family

Siblings Birth order

2.) Number of rooms in the house

**3.) Is there a separate bathroom/Toilet facilities for your family?**

Yes No

If not

Shared toilet Out side

**4.) How do you fulfill your water requirements ?**

Well water

Tap water

Spring water

Other

**5.) Do you use some methods to purify water before drinking ?**

Yes No

If yes

1. Boiled cooled water

Heat water until boiling Heat water until it feels hot at first time

B) By using a water filter

C) Treated water

**6.) Education level of parents**

Father Mother

Completed secondary education or above

Not completed secondary education

**7 ) Are your parents are employed?**

Father Mother

Employed

Not employed

**8.) Household conditions**

**a). Floor -** Cement Earthen

**b). Wall** - Cement Wood/mud

**c). Roof** - Metal laminate Board laminate

**9.) Monthly family income (SL Rupees)**

≤ 20000 > 20000

**10.) Investigation results**

Haemoglobin Height

Serum albumin Weight

BMI
